# Supplementary material for: A compressive hyperspectral video imaging system using a single-pixel detector
Source: Nat Commun. 2024 Feb 17;15:1456. doi: 10.1038/s41467-024-45856-1 (PMC10874389; doi:10.1038/s41467-024-45856-1)
Supplement: Supplementary file 1 — Supplementary Information [file 41467_2024_45856_MOESM1_ESM.pdf]

A Compressive Hyperspectral Video Imaging System Using a Single-Pixel Detector: Supplementary Information

Yibo Xu,[1*] Liyang Lu,[2] Vishwanath Saragadam,[3] and Kevin F. Kelly[3]

[1] Beijing Engineering Research Center of Mixed Reality and Advanced Display, School of Optics and Photonics, Beijing Institute of Technology, Beijing, China

[2]Google Inc., 601 N. 34th Street, Seattle, WA 98103, USA

[3]Department of Electrical and Computer Engineering, Rice University, 6100 Main St, Houston, TX 77005, USA

[*]ybxu2013@126.com

This document provides the details of the system setup and calibration of the single-doxel imager (SDI) system, multi-resolution reconstruction with STOne patterns, the process of solving the optical flow assisted 4DTV regularization problem with additional reconstruction results, compression ratio analysis, details on the deep learning reconstruction approach, and the test results of deep learning on simulation data and on test data to the primary manuscript "A Compressive Hyperspectral Video Imaging System Using a Single-Pixel Detector".

1. SDI SYSTEM SETUP AND CALIBRATION

The SDI system prototype built in lab is shown in Fig.S1a. To calibrate the spectral measurements of the SDI system, a spectral calibration lamp (Newport 6035 Hg (Ar) lamp) was used as a target to produce a typical mercury spectrum. With the spatial modulation micromirrors all fixed at the "on" state, a complete set of 256-channel spectral modulation patterns was displayed on the DMD. A spectrum was recovered from the measurements, as plotted in Fig.S1b. The three major peaks in the spectrum correspond to the 435.8 nm, 546.1 nm, and 578.2 nm emission lines of the mercury vapor. We linearly fit the wavelengths to the band numbers according to the positions of these peaks, and the result is plotted in Fig.S1c. The range of the measured wavelength is from 361 nm to 827 nm. From this spectrum, we learn that the half width at half maximum (HWHM) of the peaks is about 6 nm. Assuming the emission lines of mercury are infinitely narrow, the 6 nm HWHM determines the ultimate spectral resolution of the system. Using a narrower slit can increase the spectral resolution but will also cause loss of the light signal intensity. The slit width is a parameter of the system that can be designed depending on the number of spectral bands needed, the expected reconstruction quality, the focal lengths of other lenses in the system, the specification of the diffraction grating, etc. In the actual hyperspectral imaging with the SDI, the slit width is 600 μm. We only used 64 wavelength bands, at 7.3 nm/band for the whole spectral range from 361 nm to 827 nm, so the 6-nm HWHM closely matches the spectral sampling resolution of the system and is enough for the experiments.

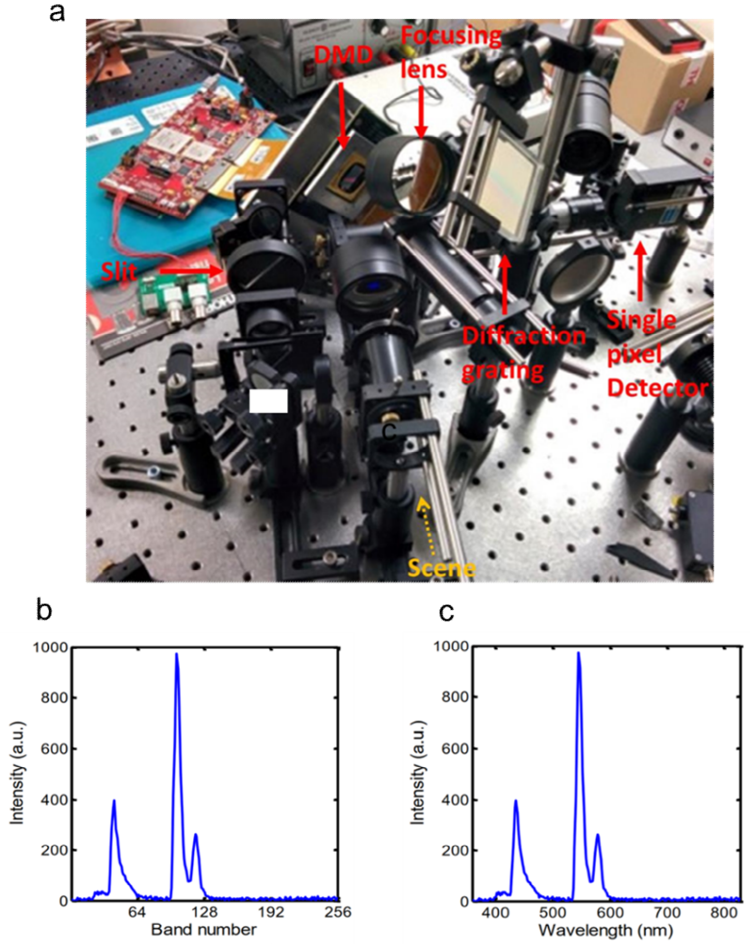

Fig. S1. (a) Photo of the SDI prototype, (b) Reconstructed spectrum of the mercury lamp, (c) Reconstructed spectrum plotted on the fitted wavelength axis.

In the experiments, 9200 spatial-spectral patterns were loaded to the DMD, with the spatial part of them at the resolution of 128 × 128 pixels and the spectral part at 64 bands. These 9200 patterns included the modulation patterns and all the complementary patterns. Each pattern covered 1024 × 1792 micromirrors on the DMD. During the measurements, the DMD displayed these patterns repetitively at the rate of 5 kHz. The ADC sampled the output of the detector at 250 kHz. Synchronized with the DMD patterns with a trigger signal, the ADC sampled 45 values for each pattern before it stopped and waited for the next pattern. These 45 sampled values were averaged to produce one measurement result for the corresponding pattern. Imaging of one hyperspectral video took about 36.8 seconds. The 9200-pattern sequence was played 20 times by the DMD, and in total 184000 measurements were taken by the SDI.

These measurement results were then processed and fed into the reconstruction algorithms to recover the hyperspectral videos of the scene.

Customized software is used to upload the designed spatial-spectral patterns to the DMD, and to control their display parameters in the measurements, such as the pattern duration, pattern sequence range, and pattern repeat times. During the measurements, the outputs of the detector are digitized by an analog-to-digital converter (ADC) and stored on the computer. The ADC also receives a trigger signal from the DMD control interface for the measurement synchronization. LabView is used to setup the signal channels of the ADC, to control the sampling rate of the detector, and to write the converted measurement results to the computer.

2. MULTI-RESOLUTION RECONSTRUCTION WITH THE STONE PATTERNS

One special property with the STOne patterns used in the SDI system is the multi-resolution structures embedded in them[1]. For a $\sqrt{N} \times \sqrt{N}$ STOne pattern formed by one of the columns from N × N matrix Φ_s, summing up every 2 × 2 pixel patch of the pattern leads to one of the two cases: a positive sum when three pixels are positive and one pixel is negative; or a negative sum when three pixels are negative and one pixel is positive, as illustrated in Fig.S2. After summing up, we get one of the $\sqrt{N/2} \times \sqrt{N/2}$ embedded low resolution STOne patterns. This down-sampling process can be repeated again and again until one of the lowest resolution 2×2 STOne patterns are obtained. In fact, a complete set of N $\sqrt{N} \times \sqrt{N}$ STOne patterns have all $2k \times 2k$ STOne patterns embedded in it, where $1 \leq k \leq log2\sqrt{N}$, giving its capability to recover at different resolutions. The ordering of the STOne pattern sequence is designed in a 'structured random' way, so that any consecutive $4k^2$ patterns in the sequence can be treated as a complete set of embedded $2k \times 2k$ STOne patterns.

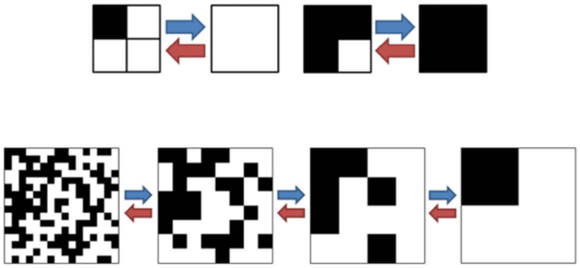

Fig. S2. The multi-resolution structure in the STOne patterns. Top row: the only two cases of down-sampling a 2 × 2 pixel patch in a STOne pattern into a pixel in the lower resolution. Bottom row: a 16 × 16 STOne pattern down-sampled to 8 × 8 STOne pattern, and further to 4 × 4 and 2 × 2 STOne pattern.

With these properties of the STOne pattern sequence, multi-resolution reconstructions can be achieved from the same set of measurements. For example, 1024 measurements with 128 × 128 STOne patterns are enough to compose a full STOne transform embedded at 32 × 32 resolution. Grayscale videos at this resolution can be calculated by a simple linear inverse transform without any iterative operations. The full 157 frames of the reconstructed 32 × 32 grayscale videos are illustrated in Fig.S3 and the calculation takes 0.5 ms per frame in Matlab. This method is useful in getting a quick look at the spatial information captured by the SDI.

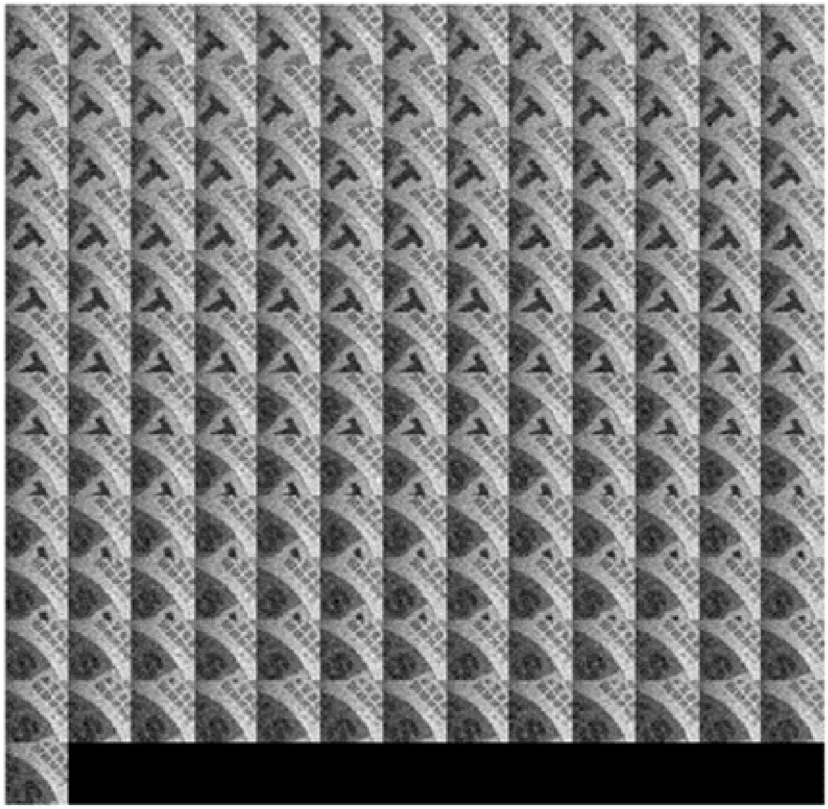

Fig. S3.157 frames of the 32 × 32 L2-reconstructed grayscale video.

The embedded low-resolution patterns can also be used to recover a low-resolution version of the hyperspectral video. The 32 × 32 × 64 low resolution hyperspectral video reconstruction, as shown in Fig.S4 takes 45 seconds per frame.

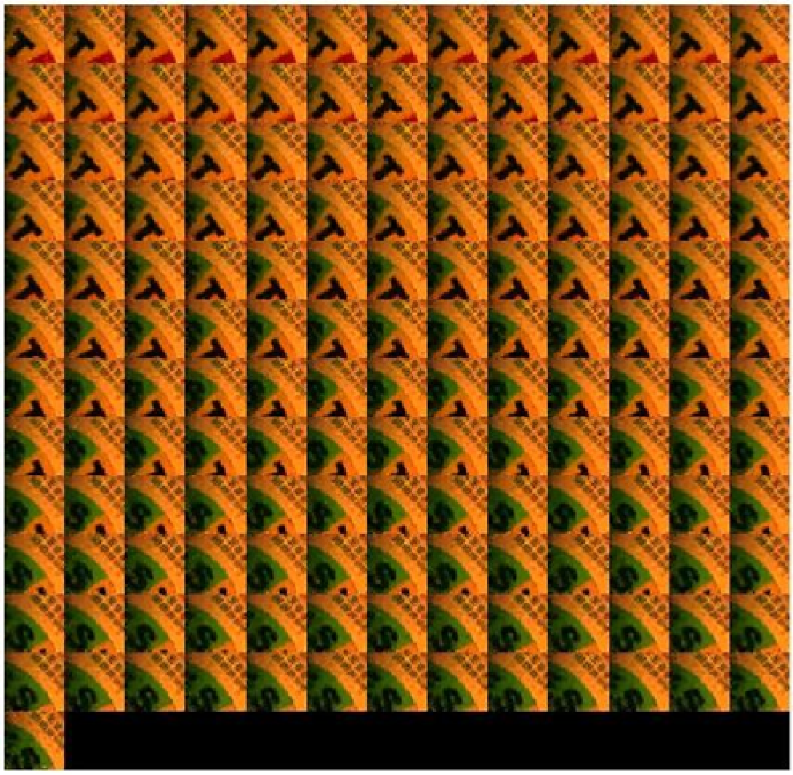

Fig. S4. 157 frames of the 32 × 32 × 64 low resolution hyperspectral video converted to RGB images

3. RECONSTRUCTION VIA OPTICAL FLOW ASSISTED 4DTV REGULARIZATION

This section presents the process of solving the optical flow assisted 4DTV regularization problem as described in Eq. (3) in the main paper. For grayscale video reconstruction, spatial measurements based on STOne patterns are calculated by summing up the two values in each spectral complementary pattern pair (see *Methods* in main paper) in the joint spectral-spatial measurements. A 3DTV-regularized algorithm[1-3] described by Eq.(S1) is solved to reconstruct the grayscale video from these calculated measurement values.

$$X = arg\ min\left|\nabla_3 X\right|,\ \ s.t. \|Y - \Psi_S X\| < \epsilon \qquad \text{(S1)}$$

$$where\ \left|\nabla_3 X\right| = \sum_{x,y,t}\left(\sqrt{\left(\frac{\partial X_{x,y,t}}{\partial x}\right)^2 + \left(\frac{\partial X_{x,y,t}}{\partial y}\right)^2} + \left|\frac{\partial X_{x,y,t}}{\partial t}\right|\right)$$

Here, X is the vectorized grayscale video, Y is a vector containing calculated spatial measurements, Ψ_S represents the sensing matrix for the spatial measurements.. Equation (S1) is solved by the Primal-Dual Hybrid Gradient (PDHG) solver

developed in Ref. 2[2].The operation of partial derivative with respect to x, y, or t in the L1 term $\left|\nabla_3 X\right|$ can be represented by a linear operator taking the difference of adjacent pixel values

In the proposed optical flow assisted 4DTV algorithm, both forward and backward optical flows are extracted between the nearby frames of the grayscale video using the algorithm developed in Ref. 4. The algorithm constructs an energy functional based on certain constraints of the optical flow model including the gray value constancy assumption, the gradient constancy assumption, and the smoothness assumption, etc. The energy functional penalizes deviations from these model assumptions and optical flow is obtained by finding a solution which minimizes the energy functional. The images are blurred using a rotationally symmetric Gaussian lowpass filter of size 7×7 with standard deviation 1 before the optical flow calculation to improve the stability of the results. Fig.S5 shows an example of the optical flows calculated between 2 frames. The first column of the figure shows 2 original images. The wheel is slightly rotating clockwise from frame 1 to frame 2. In the second column are 2 masks that indicate the pixels whose optical flow can be calculated. Due to the existence of image boundaries, occlusions, and noises, some of the pixels cannot find their matched counterparts in the other frame and are marked in red in the masks. The third column shows extracted forward and backward optical flow images. The optical flow vector field in Fig. S5 is color encoded in the same way as in Ref. 4, 5. Because of the interpolations used to achieve the sub-pixel precision of the flow vectors[4], using both forward and backward optical flows gives more accurate pixel to pixel matching between two frames. The fourth column shows two images warped according to the calculated optical flows. The warped frame 1 closely matches the original frame 2, and vice versa.

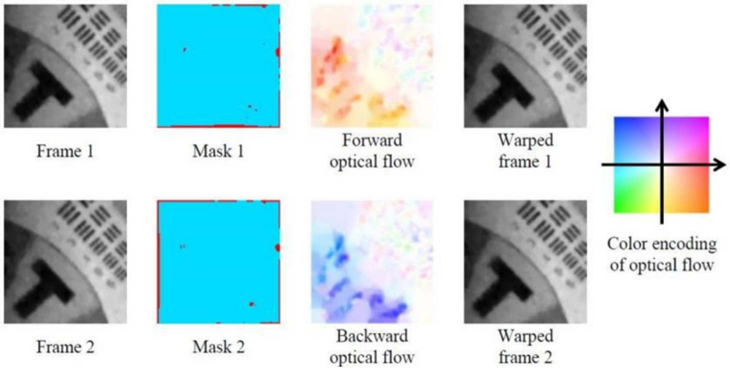

Fig. S5. Example of the optical flow calculation between two frames. From left to right are: 1. the original images, 2. the pixel masks, 3. the color encoded optical flow images, 4. the warped images using the calculated optical flows, and 5. the color encoding of optical flow.

The full 157 reconstructed 128 × 128 grayscale videos based on 3DTV algorithm and the 128 × 128 × 64 hyperspectral frames reconstructed based on optical flow assisted 4DTV algorithm are shown in Fig.S6.

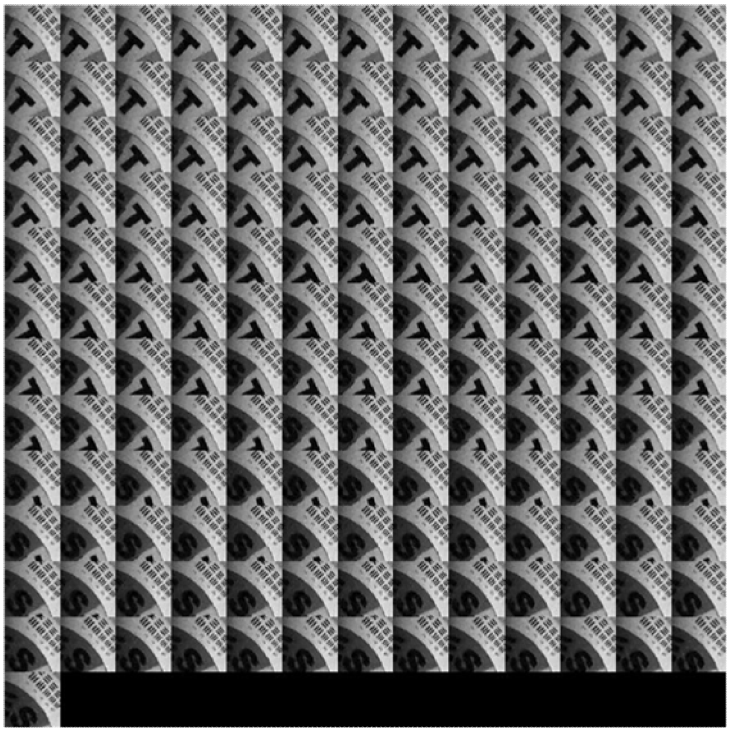

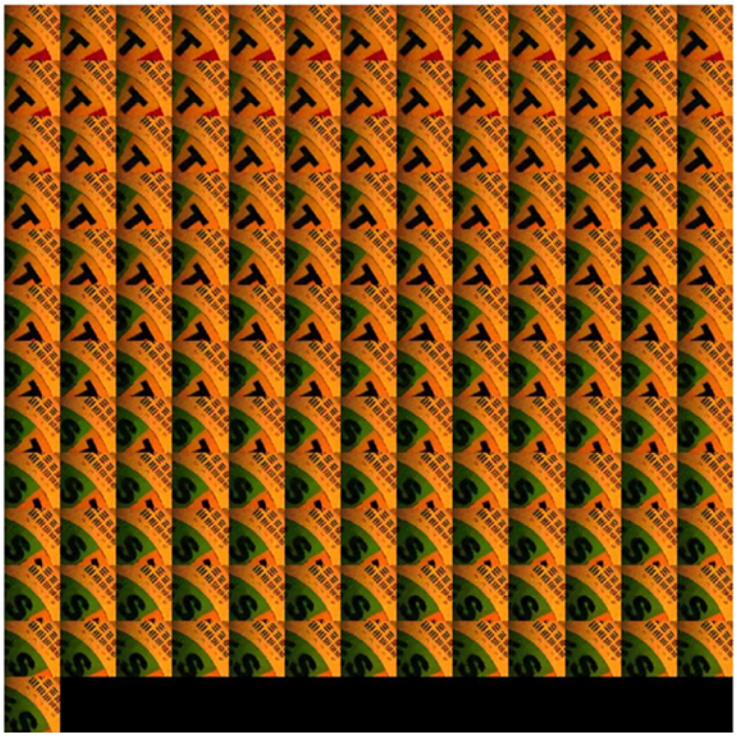

Fig. S6. Top: 157 frames of the 128 × 128 grayscale videos reconstructed based on 3DTV algorithm. Bottom: 157 frames of the 128 × 128 × 64 hyperspectral frames reconstructed based on optical flow assisted 4DTV algorithm converted to artificial RGB images.

4. RECONSTRUCTION VIA DEEP NEURAL NETWORKS

A. Testing on Simulation Data

Dataset generation. We adopt the strategy of taking publicly available hyperspectral image datasets and create a video out of each hyperspectral image by translating the image toward a certain direction. The hyperspectral datasets we used are CAVE dataset[7] and Harvard dataset[8]. The CAVE dataset consists of 32 hyperspectral images with spatial resolution 512×512. The Harvard dataset consists of 50 outdoor images captured under daylight illumination with spatial resolution 1024 × 1392. We remove 6 deteriorated images from Harvard dataset due to large-area saturated pixels. The spectral range of is from 400 nm to 700 nm for CAVE dataset and is from 420 nm to 720 nm for Harvard dataset. The spectral range of each dataset is divided into 31 spectral bands at 10 nm interval. The intensity of pixels in these datasets is rescaled to 0-1. We random select 24 images in CAVE dataset and 35 images in Harvard dataset for training and the rest for testing, respectively.

When creating the hyperspectral videos, we take a hyperspectral image from the dataset, circularly translate it along the horizontal or vertical direction for 2 pixels each time for 4 times, each time producing a new hyperspectral frame. In this manner, a video sequence consisting of 5 hyperspectral frames are obtained. For each full-size video sequence, we extract non-overlapping video blocks of size 32 × 32 × 31 × 5, indicating spatial size of 32 × 32 with 31 spectral channels and 5 temporal frames. For the CAVE dataset, we obtain 163230 of 32 × 32 × 31 × 5 hyperspectral video blocks for training the neural networks and 40808 hyperspectral video blocks for testing the neural networks. For the Harvard dataset, we obtain 196405 training blocks and 49101 test blocks. The grayscale video datasets are created by summing across the spectral dimension for each hyperspectral frame and normalizing the pixel values to 0-1, producing 32 × 32 grayscale videos. The spatial compressive measurements using the STOne patterns are taken on the videos and the reshaped vector $(\Psi_S)^T y_t$ is the input of the CNN module in the grayscale video reconstruction network. The CNN module is pretrained with the network input and output size of 32 × 32. The LSTM network takes input of a video block containing 5 grayscale video frames each reconstructed from the CNN module and outputs 5 enhanced images of size 32 × 32.

For hyperspectral frame reconstruction network, joint spectral-spatial compressive measurements are taken on each 32 × 32 × 31 hyperspectral image. Then, as described in Section 2.5 in the main paper, the vector $(\widetilde{\Phi})^T \tilde{b}_t$ is reshaped

into size of $32 \times 32 \times 31$, concatenated with the corresponding frame from the grayscale video reconstructed from the LSTM network along the spectral dimension to have size $32 \times 32 \times 32$, then used as input to the hyperspectral frame reconstruction network which outputs reconstructed hyperspectral frame of size $32 \times 32 \times 31$. For the joint spectral-spatial modulation pattern sequence, a spectral complementary pattern is inserted for every spectral pattern and a spatial spectral complementary pattern is inserted for every 32 spectral patterns. The total number of measurements used for recovering one frame for CR of 100, 25, and 10 is 316, 1270, and 3174, respectively. The measurements used for recovering nearby frames are non-overlapping.

Training Scheme. All network models are trained using Adam optimizer[9] and are implemented on NVIDIA GeForce RTX 3070 GPU with 8GB memory based on PyTorch code. Starting with the initial learning rate of 10^{-4}, we reduce the learning rate by 10% every 5 epochs. For both the grayscale video reconstruction network and the hyperspectral image reconstruction network, the loss function is the mean square error between the ground truth image and the reconstruction. For the hyperspectral image reconstruction network, because there is a residual connection from the hyperspectral network input to the output of every RC block, the network output size is designed to be $32 \times 32 \times 32$. Since the grayscale frame channel is not needed in the final hyperspectral reconstruction, we set the loss weight with respect to the grayscale frame channel to be zero.

Additional Reconstruction Results. Figure S7 and S8 illustrate additional example reconstructed hyperspectral frames using the deep learning approach for CAVE and Harvard datasets, visually demonstrating the spatial and spectral accuracy. Each image is composed of 6×6 non-overlapping tiles of $32 \times 32 \times 31$ reconstructed hyperspectral blocks converted to RGB image using CIE color mapping function. No processing is performed to smooth the boundary between blocks. The reconstructed hyperspectral videos can be found in Supplementary Video 7 and 8.

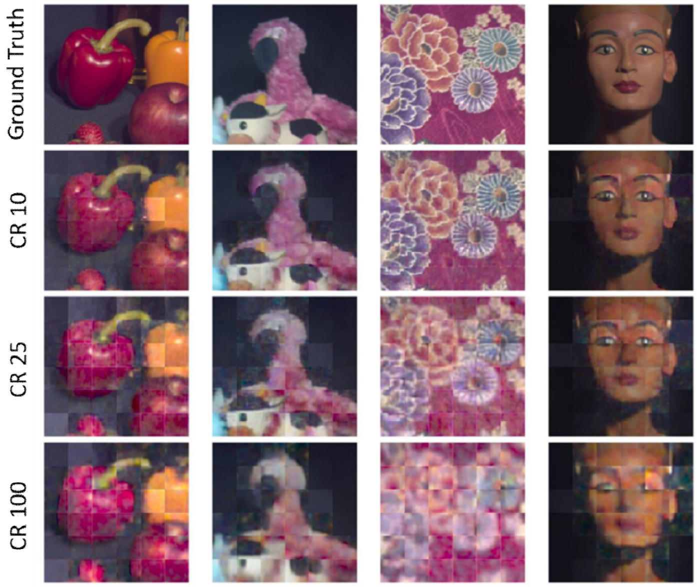

Fig. S7. Example reconstruction results of the deep learning approach of CAVE dataset. A gamma correction of gamma = 0.4 is applied on the converted RGB image to brighten darker areas. The first row is ground truth and the rest three rows are for CR of 10, 25 and 100.

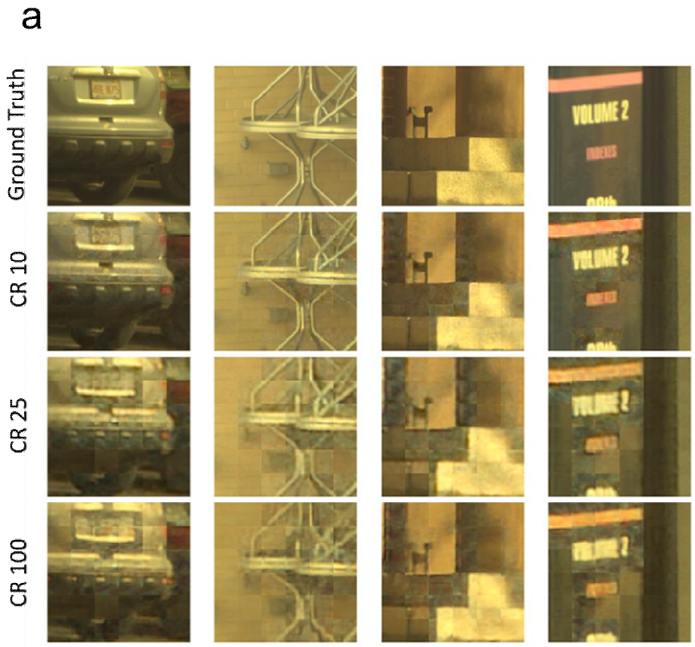

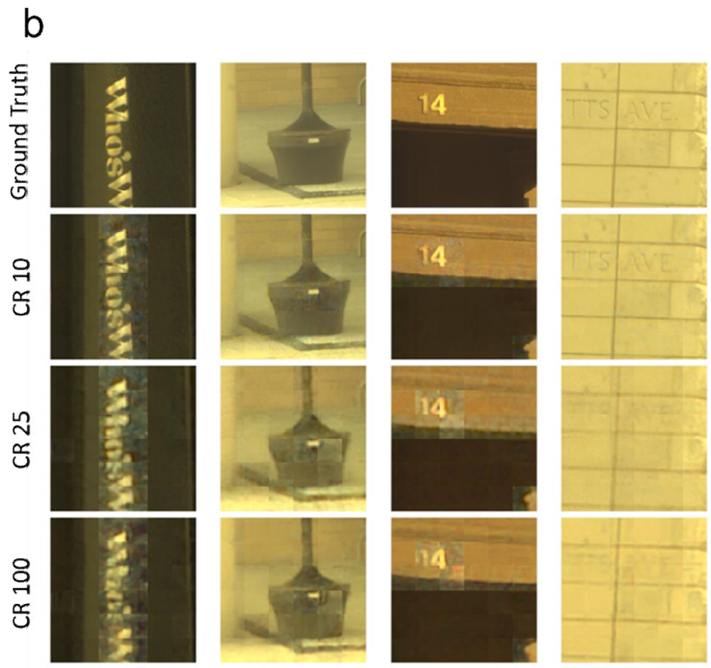

Fig. S8. Example reconstruction results of the deep learning approach of Harvard dataset. A gamma correction of gamma = 0.4 is applied on the converted RGB image to brighten darker areas. The first row in (a) and in (b) is the ground truth and the rest three rows are for CR of 10, 25 and 100.

Noise Analysis. Noise is inevitable in real scenarios. We take the network models trained without adding measurement noise and fine-tune them with training data with Gaussian or Poisson measurement noise added, then test the fined-tuned models using data with the same type of measurement noise added. The quantitative evaluation results in PSNR, SSMI, and SAM are summarized in Table S1. PSNR and SSIM are calculated between reconstructed 2D image of every spectral channel and the ground truth, then averaged across the spectral and temporal dimensions over all test data. SAM is calculated on every reconstructed 1D spectrum and its ground truth, then averaged across the spatial and temporal dimensions over all test data. With measurement noise added, performance is degraded a little bit compared to clean data but still maintain reasonable reconstruction results. When imaging in ultra high-speed mode or under extremely low-light conditions, the signal would suffer from severe noise. In these scenarios, we can use noisy data for training or fine-tune on a well-trained model to increase the robustness of the models.

	CAVE		Harvard	
	Poisson	Gaussian	Poisson	Gaussian
CR = 100	22.75/0.673/0.189	22.85/0.680/0.173	24.06/0.712/0.103	24.17/0.729/0.101
CR = 50	23.66/0.706/0.158	23.78/0.713/0.142	25.23/0.747/0.094	25.33/0.754/0.088
CR = 10	27.94/0.814/0.106	28.01/0.830/0.093	27.98/0.820/0.079	28.08/0.831/0.070

Table S1. Quantitative evaluation of PSNR(dB), SSIM, and SAM of CAVE dataset and Harvard dataset averaged over all test data after adding Poisson noise (peak value = 4095) or Gaussian noise (s = 0.04) to measurement.

B. Testing on Experimental Data

We create a simulated dataset for training neural network models which are used to reconstruct hyperspectral video from compressive measurements of a real target acquired by the SDI. Since the SDI has 64 spectral bands at 7.3 nm/band from 361 nm to 827 nm and no publicly available hyperspectral dataset has the same wavelengths, we adopt the strategy of interpolating the Harvard dataset in the spectral dimension. The Harvard dataset consists of hyperspectral images of spectral range from 420 nm to 720 nm with 31 spectral bands at 10nm interval. We linearly interpolate each of the hyperspectral images to have the same wavelength bands as the SDI between 423 nm and 715 nm, leading to 41 spectral bands. Then, the simulated hyperspectral video dataset is created by shifting and cropping the interpolated images and compressive measurements are taken on this dataset. The details are the same as described in "Dataset generation" part of Section 4A of this Supplementary Information with the only difference being 41 spectral channels instead of 31 channels. Due to GPU memory limit, the models are designed to recover hyperspectral image patches of spatial size 32 × 32 with 41 spectral bands at 7.3 nm/band from 423 nm to 715 nm. Neural network models are first trained on clean data and then fine-tuned on data with Gaussian measurement noise (s = 0.06) added. The training scheme are the same as described in the "training scheme" part of Section 4A of this Supplementary Information. Spatial-spectral modulation patterns based on the STOne patterns for spatial modulation and the pseudo-randomly permuted Walsh-Hadamard patterns for spectral modulation were used to take compressive measurements of each of the 16 blocks. The permuted Walsh-Hadamard patterns are used to provide the randomness needed for compressive sensing-based sensing and recovery and we are not aiming for multi-resolution spectral reconstruction here. The 48 × 48 permuted Hadamard matrix is first created and its first 41 columns are used to match the 41 spectral channels. Each spatial pattern covered 32 × 32 micromirrors on the DMD for a certain block with all other micromirrors put to "off" state. The spectral patterns are at 41 bands at 7.3 nm/band from 423 nm to 715 nm. To reduce motion artifact caused by the block-based measurement, the DMD is operated at a pattern rate of 15 kHz. For the joint spectral-spatial

modulation pattern sequence, a spectral complementary pattern is inserted for every spectral pattern and a spatial spectral complementary pattern is inserted for every 32 spectral patterns. The total number of measurements used for recovering one frame for CR of 100, 25, and 10 is 420, 1682, and 4208, respectively. The measurements used for recovering nearby frames are non-overlapping.

The color wheel was rotated at a lower angular velocity when measuring for lower CRs. The data of CR 25 and CR 10 are only for demonstration purpose. With appropriate neural network capacity, the block measurement strategy will not be used with the SDI and much higher CR and frame rate can be achieved. Figure S9 shows all 80 reconstructed and stitched 128 × 128 × 41 hyperspectral frames for CR 100 converted to artificial RGB images. Full videos for reconstruction of CR 100, CR 25, and CR 10 are presented in Supplementary Video 9-11.

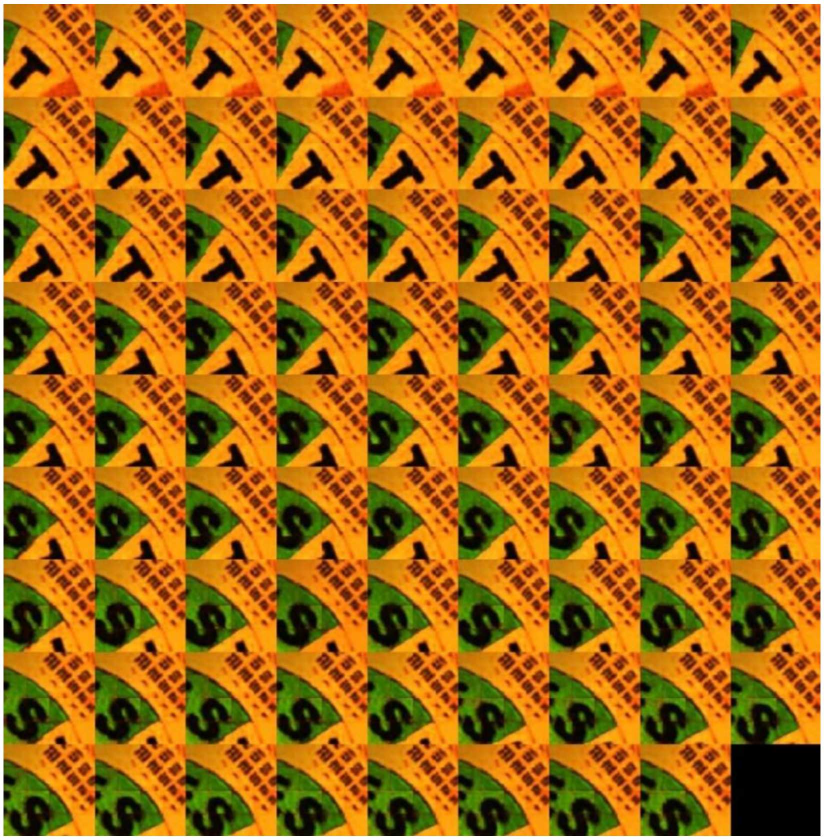

Fig. S9. 80 frames of the reconstructed 128 × 128 × 41 hyperspectral video for CR 100 converted to artificial RGB images where each frame is stitched from 32 × 32 × 41 hyperspectral patches reconstructed by the deep learning approach.

References

[1] Goldstein, T., Xu, L., Kelly, K. F. & Baraniuk, R. The stone transform: Multi-resolution image enhancement and compressive video. *IEEE Transactions on Image Processing* **24**, 5581–5593 (2015).

[2] Goldstein, T., Li, M. & Yuan, X. Adaptive primal-dual splitting methods for statistical learning and image processing. In *Advances in Neural Information Processing Systems*, 2089–2097 (2015).

[3] Baraniuk, R. G. *et al.* Compressive video sensing: Algorithms, architectures, and applications. *IEEE Signal Processing Magazine* **34**, 52–66 (2017).

[4] Liu, C. *et al. Beyond pixels: exploring new representations and applications for motion analysis*. Ph.D. thesis, Massachusetts Institute of Technology (2009).

[5] Sankaranarayanan, A. C. *et al.* Video compressive sensing for spatial multiplexing cameras using motion-flow models. *SIAM Journal on Imaging Sciences* **8**, 1489–1518 (2015).

[6] Krizhevsky, A., Sutskever, I. & Hinton, G. E. Imagenet classification with deep convolutional neural networks. *Communications of the ACM* **60**, 84–90 (2017).

[7] Yasuma, F., Mitsunaga, T., Iso, D. & Nayar, S. K. Generalized assorted pixel camera: postcapture control of resolution, dynamic range, and spectrum. *IEEE transactions on image processing* **19**, 2241–2253 (2010).

[8] Chakrabarti, A. & Zickler, T. Statistics of real-world hyperspectral images. In *CVPR 2011*, 193–200 (IEEE, 2011).

[9] Kingma, D. P. & Ba, J. Adam: A method for stochastic optimization. *arXiv preprint arXiv:1412.6980* (2014).
