# Supplementary material for: A compressive hyperspectral video imaging system using a single-pixel detector
Source: Nat Commun. 2024 Feb 17;15:1456. doi: 10.1038/s41467-024-45856-1 (PMC10874389; doi:10.1038/s41467-024-45856-1)
Supplement: Supplementary file 3 — Description of Additional Supplementary Files [file 41467_2024_45856_MOESM3_ESM.pdf]

Description of Additional Supplementary Files

Supplementary Video 1:
Reconstructed hyperspectral video including 157 frames of spatial size 128x128 with 64 spectral bands reconstructed using the optical flow assisted 4DTV algorithm. Hyperspectral frames are converted to artificial RGB images.

Supplementary Video 2:
Reconstructed hyperspectral video showing individual bands including157 frames of spatial size 128x128 with 64 spectral bands reconstructed using the optical flow assisted 4DTV algorithm. Each of the 64 bands are displayed converted to artificial RGB images.

Supplementary Video 3:
Reconstructed grayscale video including157 frames of spatial size 128x128 reconstructed using the 3DTV algorithm.

Supplementary Video 4:
Reconstructed low-resolution hyperspectral video including 157 frames of spatial size 32x32 with 64 spectral bands reconstructed using the embedded low resolution STOne patterns and the optical flow assisted 4DTV algorithm. Hyperspectral frames are converted to artificial RGB images.

Supplementary Video 5：
Reconstructed low-resolution hyperspectral video displaying individual bands including 157 frames of spatial size 32x32 with 64 spectral bands reconstructed using the embedded low resolution STOne patterns and the optical flow assisted 4DTV algorithm. Each spectral channel is displayed converted to artificial RGB images.

Supplementary Video 6：
Reconstructed low-resolution grayscale video inclduing157 frames of spatial size 32x32 reconstructed via the embedded low resolution STOne patterns and using direct inversion.

Supplementary Video 7：
Reconstructed hyperspectral videos of the CAVE dataset of spatial size 192x192 with 31 spectral bands. Each video frame is stitched from 6x6 tiles of hyperspectral patches.

Supplementary Video 8：
Reconstructed hyperspectral videos of the Harvard dataset of spatial size 192x192 with 31 spectral bands. Each video frame is stitched from 6x6 tiles of hyperspectral patches.

Supplementary Video 9：
Reconstructed hyperspectral video of experimental data using deep learning method for compression ratio of 100. Each frame is stitched from 4x4 tiles of reconstructed hyperspectral patches.

Supplementary Video 10：

Reconstructed hyperspectral video of experimental data using deep learning method for compression ratio of 25. Each frame is stitched from 4x4 tiles of reconstructed hyperspectral patches.

Supplementary Video 11：

Reconstructed hyperspectral video of experimental data using deep learning method for compression ratio of 10. Each frame is stitched from 4x4 tiles of reconstructed hyperspectral patches.
